# Supplementary material for: Menopausal hormone therapy: Characterising users in an Australian national cross-sectional study
Source: PLoS One. 2021 Aug 11;16(8):e0253725. doi: 10.1371/journal.pone.0253725 (PMC8357145; doi:10.1371/journal.pone.0253725)
Supplement: S1 Table — (DOCX) [file pone.0253725.s001.docx]

**Supplementary Table 1: Associations between socio-demographic characteristics and menopausal hormone therapy use after 2002, between short-term users† (<5 years) and long-term users (≥5 years)‡ compared to never users, among Australian women aged 50-69 years old.**

| **Characteristic** | **Never-user**  **n= 1214** | **Short-term users**  **N=255** | **OR (95%CI)^** | **Long-term users**  **N=85** | **Adjusted OR (95%CI)^** | ***p*-value 1*,**  ***p*-value 2,**  ***p*-value 3** |
| --- | --- | --- | --- | --- | --- | --- |
| **Age group (years)** |  |  |  |  |  |  |
| 50-54 | 692 (83) | 130 (16) | Ref. | 16 (02) | Ref. | 0.051 |
| 55-59 | 472 (74) | 108 (17) | 1.28 (0.94-1.72) | 59 (09) | 6.28 (3.41-11.54) | <0.001 |
| 60-64 | 50 (65) | 17 (22) | 2.02 (1.07-3.80) | 10 (13) | 9.34 (3.61-24.12) | <0.001 |
|  |  |  |  |  |  |  |
| **Country of Birth** |  |  |  |  |  |  |
| Australia | 917 (79) | 184 (16) | Ref. | 59 (5) | Ref. | 0.415 |
| English-speaking country | 164 (73) | 44 (20) | 1.31 (0.88-1.95) | 17 (8) | 1.83 (0.95-3.52) | 0.192 |
| Non-English speaking country | 133 (79) | 27 (16) | 1.09 (0.66-1.79) | 09 (05) | 1.15 (0.48-2.72) | 0.647 |
|  |  |  |  |  |  |  |
| **Highest qualification** |  |  |  |  |  |  |
| No school | 64 (70) | 17 (18) | Ref. | 11 (12) | Ref. |  |
| High school/ trade/ apprenticeship | 425 (79) | 86 (16) | 0.67 (0.36-1.28) | 27 (05) | 0.29 (0.12 – 0.73) | 0.648 |
| Certificate diploma | 330 (79) | 72 (17) | 0.71 (0.37-1.39) | 18 (04) | 0.25 (0.09 - 0.67) | 0.041 |
| University degree or higher | 395 (78) | 80 (16) | 0.66 (0.33-1.29) | 29 (06) | 0.30 (0.11-0.80) | 0.296 |
|  |  |  |  |  |  |  |
| **Marital status** |  |  |  |  |  |  |
| Single-never married | 93 (82) | 14 (12) | Ref. | 07 (06) | Ref. | 0.487 |
| Married /partnered | 932 (77) | 207 (17) | 1.38 (0.72-2.66) | 69 (06) | 1.00 (0.37- 2.73) | 0.594 |
| Single-divorced, separated, widowed | 189 (82) | 34 (15) | 1.14 (0.55-2.38) | 09 (04) | 0.66 (0.20 - 2.14) | 0.716 |
|  |  |  |  |  |  |  |
| **SEIFA quintile** |  |  |  |  |  |  |
| 1 - lowest | 154 (79) | 30 (15) | Ref. | 11 (06) | Ref. |  |
| 2 | 210 (80) | 41 (16) | 0.88 (0.51 -1.52) | 11 (04) | 0.63 (0.24 - 1.67) | 0.750 |
| 3 | 228 (76) | 59 (20) | 1.17 (0.69-1.99) | 13 (04) | 0.63 (0.24 - 1.66) | 0.561 |
| 4 | 242 (78) | 51 (17) | 0.93 (0.54-1.60) | 17 (06) | 0.83 (0.33 – 2.11) | 0.445 |
| 5 - highest | 380 (78) | 74 (15) | 0.95 (0.55-1.63) | 33 (07) | 1.09 (0.44 – 2.72) |  |
|  |  |  |  |  |  |  |
| **ARIA plus** |  |  |  |  |  |  |
| Major City | 742 (78) | 149 (16) | Ref. | 57 (06) | Ref. | 0.514 |
| Inner Regional | 293 (77) | 70 (18) | 1.24 (0.86-1.78) | 19 (05) | 0.84 (0.44 - 1.58) | 0.843 |
| Outer regional/  remote/very remote | 179 (80) | 36 (16) | 1.13 (0.70-1.80) | 09 (04) | 1.01 (0.41 – 2.47) | 0.530 |
|  |  |  |  |  |  |  |
| **Income (per year)** |  |  |  |  |  |  |
| ≤ $25,000 | 317 (79) | 57 (14) | Ref. | 29 (07) | Ref. | 0.273 |
| $25,001-$100,000 | 337 (78) | 79 (18) | 1.32 (0.87-1.98) | 17 (04) | 0.55 (0.27 - 1.13) | 0.110 |
| ≥ $100,001 | 290 (75) | 71 (18) | 1.43 (0.90-2.25) | 28 (07) | 1.16 (0.57 - 2.38) | 0.071 |
| Prefer not to answer | 270 (82) | 48 (15) | 0.96 (0.62-1.53) | 11 (03) | 0.35 (0.16 – 0.77) |  |
|  |  |  |  |  |  |  |
| **Occupation** |  |  |  |  |  |  |
| Employed | 930 (78) | 202 (17) | Ref. | 54 (05) | Ref. | 0.853 |
| Unemployed | 35 (80) | 6 (14) | 0.83 (0.33-2.09) | 3 (07) | 1.15 (0.29 – 4.51) | 0.379 |
| Not in labour force (e.g. retired) | 249 (77) | 47 (15) | 0.92 (0.62-1.35) | 28 (09) | 1.51 (0.85 – 2.68) | 0.307 |

† Short term users: initiated MHT on or after 2002, used MHT for less than 5 years and stopped use before survey completion to reduce the possibility of becoming a long-term user. Women who started and stopped MHT in 2002 were excluded.

‡ Long-terms users: started MHT from 2002 onwards and used MHT for 5 or more years. Some would have stopped MHT before survey completion and others would be current users at the time of survey completion.

^ Adjusted ORs obtained from multinomial logistic regression model. The dependent variable in the regression model was MHT use after 2002 (yes vs no) while the independent variables were the characteristics listed in Supplementary Tables 1, 2 and 3.

* p-value 1: global p-value for the relationship with short-term (<5 years) MHT use vs never use; p-value 2: global p-value for the relationship with long-term (≥5 years) MHT use vs never use; p-value 3: global p-value for whether adjusted OR estimates differ between short and long-term users.
